# Excessive Hæmorrhage from Extracting a Tooth

**Published:** 1848-04

**Authors:** 


From the Dental Recorder.

### EXCESSIVE HÆMORRHAGE FROM EXTRACTING A TOOTH.

Case.—After filling several teeth for Mrs. L———, I advised the extraction of two fangs, the remains of the anterior and superior bicuspides, to which she consented, and they were removed without any difficulty. At the time of the operation, the bleeding was not greater than usual, and she soon after left the office. Twenty-four hours after, she called again, and informed me that the gum on one side, from which the longest root had been extracted, had continued to bleed ever since the operation. During the evening, she had applied an astringent tincture, procured from a dentist near by, on pledgets of cotton, but without producing any effect on the hæmorrhage which continued as before. Alum was also tried several times in the night in the same way, but produced no effect. She informed me that she had experienced the same difficulty when having teeth extracted before, and that her child had also bled profusely from having a temporary tooth removed.

I learned also that a few years before she had suffered greatly from hæmorrhage after having an abortion, from which she narrowly escaped with her life. These facts showed plainly the existence of a hæmorrhagic diathesis, in which there is generally a want of strong and healthy action in the vascular system, especially in the capilary vessels.

The gums were in a soft and spongy condition, and bled freely on slight irritation. From all these indications, I formed the opinion that the bleeding was kept up from a want of contractile power in the mouths of the ruptured vessels, and not from excessive action.

One of the oldest pathological distinctions, and I believe a correct one among hæmorrhages, is the *active* and the *passive:* the former exists when the vascular system is in an undue state of excitement. Of this character are the critical hæmorrhages which often take place in fevers and other acute diseases. At other times hæmorrhagy exists with a state of general constitutional debility, and arises from causes that obviously weaken the tone of the system, as is well exemplified in some of the cases of menorhagia; it is then denominated *passive.* Of this character I judged the hæmorrhage to be in the present case. The indication, therefore, seemed to be to apply a stimulus, which I did, and had the satisfaction to see the bleeding stop in a few moments. After wiping away the coagula from the socket, I applied a pledget of cotton dipped in the tincture of anthemis pyrethrum or pelatory of Spain. The cotton came away in a short time, and there was no recurrence of the bleeding. This practice will, I believe, generally be found successful in cases of this kind, which often alarm the patient, and give the dentist considerable trouble and vexation. A.

